# The Paradoxical Role of NKG2D in Cancer Immunity

**DOI:** 10.3389/fimmu.2018.01808

**Published:** 2018-08-13

**Authors:** Sam Sheppard, Amir Ferry, Joana Guedes, Nadia Guerra

**Affiliations:** ^1^Department of Life Sciences, Imperial College London, London, United Kingdom; ^2^Memorial Sloan Kettering Cancer Center, Zuckerman Research Center, New York, NY, United States

**Keywords:** NKG2D, cancer, hepatocellular carcinoma, inflammation, CD8^+^ T cells, natural killer cells

## Abstract

The activating receptor NKG2D and its ligands are recognized as a potent immune axis that controls tumor growth and microbial infections. With regards to cancer surveillance, various studies have demonstrated the antitumor function mediated by NKG2D on natural killer cells and on conventional and unconventional T cells. The use of NKG2D-deficient mice established the importance of NKG2D in delaying tumor development in transgenic mouse models of cancer. However, we recently demonstrated an unexpected, flip side to this coin, the ability for NKG2D to contribute to tumor growth in a model of inflammation-driven liver cancer. With a focus on the liver, here, we review current knowledge of NKG2D-mediated tumor surveillance and discuss evidence supporting a dual role for NKG2D in cancer immunity. We postulate that in certain advanced cancers, expression of ligands for NKG2D can drive cancer progression rather than rejection. We propose that the nature of the microenvironment within and surrounding tumors impacts the outcome of NKG2D activation. In a form of autoimmune attack, NKG2D promotes tissue damage, mostly in the inflamed tissue adjacent to the tumor, facilitating tumor progression while being ineffective at rejecting transformed cells in the tumor bed.

Natural killer (NK) cells were discovered more than four decades ago, initially described as spontaneous cytolytic effector cells, operating rapidly without the T and B lymphocyte requirement for antigen presentation (1). The discoveries of multiple stimulatory and inhibitory NK cell receptors have helped refine our understanding of NK cell target recognition. These included the NKG2 family of transcripts identified by Houchins and colleagues, which encode type II integral membrane proteins (2–4). Unlike the majority of NKG2 receptors that form heterodimers with CD94, NKG2D forms a homodimer transmembrane C-type lectin-like receptor. NKG2D is highly conserved and although first identified on NK cells is expressed on both innate and adaptive lymphocytes, including NK cells, αβ T cells, γδ T cells, iNKT cells, and innate lymphoid cells (5–7). Identification of NKG2D ligands and their expression in response to cellular stress by the group of Thomas Spies revealed the critical link between NKG2D and stress-induced tissue damage (8–10). This potent activating receptor has been and remains the subject of intense research in cancer, infection, and autoimmunity.

NKG2D ligands are cell surface proteins structurally related to major histocompatibility complex (MHC). In humans, these ligands are the MHC class I-related chain A and B (MICA, MICB) proteins and the six unique long 16 (UL-16)-binding proteins (ULBP1-6) (11). Their counterparts in mice are the retinoic acid early inducible gene-1 (RAE-1α-ε), minor histocompatibility H60a-c, and murine UL16-binding protein-like transcript 1 (MULT1) proteins. Our understanding of ligand regulation is far from complete with continuing discovery of regulatory mechanisms and pathways. Induction and upregulation of NKG2D ligands result from various stress signals including infection, DNA damage, heat shock, and hyperproliferation (12, 13). Regulation of ligand expression appears to occur predominantly at the transcriptional level (14–17); however, posttranscriptional (18–20) and epigenetic regulation have also been reported (21, 22). NKG2D ligands can be released from the cell surface by proteases of the matrix and desintegrin metalloproteinases families (MMP and ADAM) (23–25), alternatively, ligands can be secreted or released as exosome-bound ligands. Soluble or exosome bound ligands are often detected in the serum of patients with advanced cancer (26) and autoimmune diseases (27–29).

## NKG2D in Tumor Immunity

A large body of experimental evidence established that NKG2D plays an important role in the surveillance of tumors by the immune system. NKG2D-dependent elimination of tumor cells that express at least one cognate ligand has been well documented *in vitro* (5, 8, 30, 31) and using *in vivo* models of transplanted tumors (16, 32–34). Direct evidence supporting a role for NKG2D in tumor surveillance came from studying tumor development in gene-targeted mice that lack NKG2D and carry transgenes that trigger tumorigenesis (35), mice with transgenic expression of human NKG2D ligand (36), and in a model of antibody-mediated NKG2D neutralization (37). Indirect evidence comes from model studies of failed tumor surveillance associated with the downregulation of NKG2D on NK cells. Constitutive expression of RAE-1ε led to systemic NKG2D downregulation that correlated with increased tumor burden in skin cancer (38) and an increased incidence of B cell lymphomas (39).

Expression of NKG2D ligands has been observed in human cancers arising from a variety of tissues. Variable expression of MICA, MICB, and ULBP1-3 ligands was observed in hematopoietic malignancies, including acute and chronic leukemias of lymphoid and myeloid origins (40), in addition to solid tumors such as neuroblastoma (41), colorectal (42), ovarian (43), cervical (44), breast (45), pancreatic (46), melanoma (47–49), and gastric cancers (50). One common feature is the heterogeneity in ligand expression between cancer types and individuals (42, 45, 47, 51), which hinders the prognostic value of NKG2D ligands in clinical assessment. Indeed, several reports have highlighted the paradoxical relationship between ligand expression and patient outcome. Studies of colorectal (42), cervical (44), and nasopharyngeal carcinoma (52) correlated high levels of surface ligand expression with improved disease-free survival, supporting the role of NKG2D in antitumor immunity. Conversely, high levels of cell surface ligand associated with poor prognosis in breast cancer (53), lung (54), and ovarian cancers (43, 55) suggest a failure in NKG2D-mediated tumor surveillance and/or that high levels of surface ligand drives disease progression. Specifically, Li and colleagues showed that high expression of ULBP2 detected by immunohistochemistry in 82 ovarian cancer patients correlated with less intraepithelial infiltration of T cells and poor prognosis (55). The authors found no correlation between the presence of soluble ligands and increased tumor stage undermining a role for soluble ligands in disease progression (55). McGilvray and colleagues corroborated the poor prognosis in ovarian cancer using a larger cohort of patients where expression of high levels of ULBP-1-5 correlated with decreased survival, whereas MICA expression did not correlate with disease progression (43). Madjd and colleagues studied a large cohort of 530 invasive breast cancer patients and showed that high intensity of MICA expression correlated with poor prognosis. In 50 cases studied for CD56 expression, the authors found absent or low NK cell infiltrate, yet, that did not correlate with MICA expression or prognosis (53). In non-small cell lung carcinoma, Chen and colleagues observed that 62% of 222 patients expressed high levels of MICA, which correlated with a decrease in median survival (54). Discrepancies might be accounted for by the variation in the nature of the ligand(s), i.e., their binding affinity to NKG2D (56, 57). de Kruijf et al. showed that ULBP-2 and major histocompatibility class I-related chain (MICA/B) expression, but not ULBP-1,3,4 or 5, correlated with longer relapse-free survival in breast cancer patients (45). The functional outcome of ligand variety on NK cell activation was recently evidenced using super-resolution microscopy (58). MICA and ULBP2 differentially affect NKG2D nanoscale reorganization at the NK cell membrane and subsequent NK cell activation. Binding to ULBP2, but not MICA, caused NKG2D nanoclusters to coalesce with the IL-2/IL-15 receptor beta subunit, leading to a greater production of IFN-γ (58). The function of NKG2D itself can also differ with different NKG2D (*KLRK1*) gene polymorphisms and associate with susceptibility to cancer. The low cytotoxic activity related to the NKG2D haplotype *LNK1/LNK1*, found in one-third of the general population, was associated with increased cancer development (59).

Many factors that constitute the tumor microenvironment (TME) impact the efficacy of the NKG2D-mediated antitumor response and with-it clinical outcome. These will be discussed throughout this paper, they include: (i) the presence of proteases that shed cell surface ligands, (ii) the quantity and quality of immune cells infiltrating tumors and (iii) the presence of cytokines that regulate the expression of NKG2D receptor and ligands. Clonal evolution of tumors is an additional variable likely to affect each of these parameters over the course of disease progression.

## NKG2D/NKG2D-Ligand Regulation in Complex and Diverse Tumor Environments

NKG2D ligand expression can be downregulated from the cell surface due to hypoxia and intracellular retention by the CEACAM1 tumor-associated antigen, reducing sensitivity to NK cell lysis (60–62). Also, metalloproteases present in the TME have a negative impact on tumor surveillance by releasing soluble ligands known to downregulate NKG2D on NK and CD8^+^ T cells (41, 63–67). As a consequence, the presence of soluble ligands in the sera of cancer patients is often associated with poor prognosis (47, 68, 69), including in patients treated with checkpoint blockade therapy as recently shown in metastatic melanoma (70). Using an antibody to block ligand shedding, preclinical studies demonstrated the antitumor potential of NKG2D in rejecting metastases in mouse and humanized mouse models of MICA-transduced transplanted B16-F10, CT26, and A2058 mouse and human tumors (71). Nonetheless, the presence of soluble ligands is not always associated with NKG2D downregulation and impaired antiviral (72) and antitumor activities. Indeed, NKG2D expression on NK cells from stage IV melanoma patients did not significantly differ from age-matched healthy controls despite the presence of high sMICA (47). Soluble MICB present in the sera of patients with gastrointestinal tumors failed to alter NKG2D expression on NK cells *in vitro* (73). In ovarian cancer, high levels of sMICA and sULBP2 present in ascites samples did not correlate with a decreased expression of NKG2D on T cells or NK cells (74). Tumor-cell derived soluble ULBP2 did not induce NKG2D downregulation on NK cells *in vitro* as opposed to membrane-bound ULBP2 (75). Also, animal studies revealed that the secreted form of MULT1, the mouse equivalent of ULBP-1 with a unique high affinity, does not downregulate NKG2D but rather favors tumor rejection by stabilizing NKG2D expression and preventing NK cell desensitization induced by RAE-1 on myeloid cells (76).

An additional layer of complexity rests on the fact that non-tumor cells, including immune cells, can upregulate NKG2D ligands and negatively regulate NKG2D expression (77). mRNA transcripts for human and mouse NKG2D ligands are widely detected in healthy tissues (78), and protein expression is detected in low amounts in the intestinal tract (9), liver (79, 80), bronchial epithelial cells (81), endothelial cells (82), and on myeloid cells (83). In fact, contact-dependent and independent interactions between myeloid cells and NK cells have been shown to either enhance (84, 85) or impair (76, 86, 87) NK cell-mediated antitumor activity. Interestingly, NK cells express ADAM17, which can cleave NKG2D ligands from the cell membrane. Furthermore, in response to IL-15 + IL-12 + IL18, human NK cells can produce soluble NKG2D ligand in an NKG2D- and ADAM17-dependent manner (88). Thus, NK cells may play a role in shaping the NKG2D ligand environment.

Several cytokines present in the TME and adjacent tissue positively or negatively influence the expression of NKG2D receptor and ligands. IFN-γ was shown to decrease MICA and, in some cases, ULBP2 expression on human melanoma cell lines (89); Type 1 interferons can reduce H60 expression in the methylcholanthrene (MCA)-induced mouse model of fibrosarcoma (90) or increase MICA/B surface expression on human pancreatic cancer cell lines (91). TGF-β downregulates NKG2D on NK cells and CD8^+^ T cells in cancer patients (79, 92, 93) and NKG2D ligands on cancer cells (94, 95). This was shown in glioma patients with heterogeneous expression of NKG2D ligands where TGF-β selectively downregulated MICA and ULBP2 transcription, but not MICB, ULBP1, and ULBP3 (95). IL-15 is well recognized for its ability to enhance NKG2D expression and activation (58, 96) whereas IL-21 that shares the common receptor γ chain operates in a context-dependent manner. Culturing human primary NK and CD8^+^T cells with IL-21 was shown to downregulate surface expression of NKG2D and DAP10 transcription (97). However, a previous study showed an opposing role for IL-21 (98) in its capacity to enhance NKG2D-dependent tumor rejection in mice. This may be explained by additional signaling *via* mouse DAP12, which can pair with mouse NKG2D but not human NKG2D, highlighting the species-specific nature of NKG2D signaling (99). Thus, in model studies, it is possible that depending on the cytokine milieu, preferential activation of DAP10 versus DAP12 guides the outcome of NK cell activation toward cytokine release and/or cytotoxic activity (100). This difference in signaling between species is restricted to innate lymphocytes since NKG2D only signals *via* DAP10 in mouse CD8^+^ T cells (34). Collectively, these studies highlight the heterogeneous expression of cell-bound and soluble forms of NKG2D ligands across cancer types and individuals, which ultimately challenges its benefit as a prognostic biomarker.

## Hepatocellular Carcinoma (HCC)-NKG2D Ligand Expression in a Typical Inflammation-Driven Cancer

The majority of HCCs develop from a background of chronic inflammation, which is now recognized as a hallmark of cancer (101). Persistent liver damage leading to hepatocyte death can trigger the production of IL-1α inducing the expression of TNFα, which in turn upregulates IL-6 (102, 103). In addition to its ability to modulate the immune response, IL-6 can also induce the production of hepatocyte growth factor, thereby stimulating hepatocyte proliferation to compensate for hepatocyte death (104, 105). Damaged hepatocytes will harbor genetic mutations that may either drive tumorigenesis (106, 107) or result in further cell death, creating a positive feedback loop (108, 109).

Chronic inflammation that precedes HCC is most commonly caused by chronic hepatitis B (HBV) or hepatitis C (HCV) infection, particularly in the developing world, although increasing numbers of cases develop from non-viral hepatitis induced by hepatic stress resulting from the excessive consumption of alcohol and or a high calorie diet (110, 111). The importance of the NKG2D axis in clearing liver infection has been well documented. In HCV or HBV infected patients, high levels of NKG2D expression on intrahepatic T cells (112), NK (79, 113–115), and iNKT cells (116) have been reported. During the course of HCV infection, MICA/B expression is elevated compared to healthy individuals (79) and HCV proteins were shown to enhance cell surface expression of ULBP-1 on human immortalized hepatocytes (117). Expression of MICA and MICB on transformed hepatocytes was also observed on hepatoma cell lines, HCCs (80, 118), and carcinoma cell lines (119). However, as seen in other tissues/diseases, expression of NKG2D ligands in hepatitis-associated conditions is heterogeneous, and there is still conflicting evidence as to the beneficial versus deleterious role of NKG2D in hepatitis. Various *MICA* alleles were shown to be over- or under-represented in infected individuals that develop HCC. Specifically, MICA 251 Gln, MICA 175 Gly, MICA 129 Met, or a promoter region variant MICA rs259654A are significantly more prevalent in HBV-infected individuals that progress to HCC than in HBV-infected individuals who developed liver cirrhosis, but not HCC (120). Fang and colleagues demonstrated in a cohort of 96 HCC patients that low frequency of MICA/B surface expression correlated with high tumor grade and reduced overall survival (121). However, in a study including 47 HCC patients, Kamimura and colleagues demonstrated ULBP1 to be expressed in well-differentiated and moderately differentiated HCC, but absent from poorly differentiated HCC, which significantly correlated with early recurrence but not with overall survival (119). In this study, MICA was mainly expressed on endothelial cells and ULBP2-4 were not expressed (119). Soluble MICA/B have been reported to be elevated in patients with viral hepatitis compared to healthy individuals (79, 122) and correlated with markers of liver damage such as serum ALT and AST (120). Conversely, in HCV-infected patients, the SNP rs2596538 allele A has been linked to lower serum levels of sMICA and to the progression from hepatitis to HCC (123). Also, there is evidence that the NKG2D axis plays a role in non-viral autoimmune hepatitis. Patients diagnosed with non-alcoholic fatty liver (NAFL) or non-alcoholic steatohepatitis (NASH) showed significantly increased expression of MICA and MICB on hepatocytes. The increase in MICA/B mRNA levels correlated with decreased liver function, increased fibrosis, and hepatocyte apoptosis (124).

Together, these studies attest to the relevance of the NKG2D pathway in regulating immune responses in the liver and illustrate how sustained expression of NKG2D ligands can exacerbate liver tissue damage. In our view, this supports the idea that chronic NKG2D activation during viral or autoimmune inflammation could be a common feature of cancer driven by inflammation. Indeed, there is a growing body of evidence implicating the NKG2D/NKG2D ligand axis as a driver of inflammatory disorders *via* direct targeting of healthy tissues expressing NKG2D ligands and/or *via* secretion of cytokines that exacerbates the initial inflammation (125–127). We hypothesize that NKG2D could act as a dual player in cancer immunity in a context and time-dependent manner. We postulate that previously beneficial inflammatory responses against early neoplastic lesions or infectious agents contribute to tissue injury and tumor progression over time (128).

To test this hypothesis, we chose to focus on a model of liver cancer, diethylnitrosamine (DEN)-induced HCCs, due to the established causative link between chronic inflammation and tumorigenesis. A key feature of the DEN-induced HCC mouse model is the slow, physiologic development of autochthonous tumors over a 9- to 15-month period. This mimics important characteristics of humans advanced HCC, even though it does not recapitulate the liver fibrosis often associated with liver cancer. First, the DEN-induced HCC model mimics the heterogeneity of human HCC (129), demonstrating a high variation in the incidence rate of mutations in the B-raf and H-ras genes between mouse strains (130). Also, the upregulation of glypican-3 on transformed hepatocytes and other markers (glutamine synthetase and heat shock protein 70) in this model supports its relevance to recapitulate human HCC (131). Second, HCC comparative genomic studies have shown that DEN-induced HCC displays gene expression profiles with characteristics similar to those of human tumor biopsies taken from patients with a poor prognosis (132), including inflammatory signatures such as TNFα and IL-6 and activation of nuclear factor-κB (105, 133). Third, the DEN model also recreates the HCC gender bias observed in humans, where male:female incidence averages between 2:1 and 4:1 in most populations (105, 110). Finally, the DEN-induced HCC model recapitulates similar features described in human hepatitis and HCC including: (i) elevated levels of NKG2D and NKG2D ligands, (ii) expression of NKG2D ligands on healthy tissue, (iii) evidence for a high CD8^+^ T cell infiltrate that correlates with tissue damage and HCC, and (iv) potential dysfunction in NK cell subsets. Although human and mouse NKG2D ligands differ (99), they have both evolved to be capable of binding strongly to NKG2D (134). Molecular modeling showed that the ligand-binding site of NKG2D is highly conserved between both species, with mouse NKG2D being capable of binding all the human ligands (135). Nonetheless, MULT-1 displays a high affinity for NKG2D that is unparalleled by human ligands.

## The Paradigm Shift: NKG2D Contributes to Tumor Progression in HCC

We treated NKG2D-deficient (*Klrk1^−/−^*) and sufficient (*Klrk1^+/+^*) mice with DEN to induce liver damage and study the progression of HCC, the expression of NKG2D ligands, and the immune composition of the liver tissue. Compared to *Klrk1^−/−^* mice, wild-type mice displayed reduced survival and increased tumor burden assessed according to three criteria: liver/body weight ratio, maximal tumor size, and tumor load (136). Histopathology analyses showed similar incidence of adenomas, benign nodules, and HCC in both genotypes, with malignant HCC developing in more than 70% of DEN treated mice by the time they reached the end point. These findings indicate that NKG2D did not impact tumor incidence in this model but significantly accelerated HCC progression once established (136). RAE-1 was highly expressed on tumors developing in both wild-type and *Klrk1^−/−^* mice indicating that tumor progression was not the consequence of escape *via* ligand editing.

In agreement with our findings, various model studies support the idea that NKG2D-expressing cells have the potential to drive, rather than resolve hepatitis. In the apolipoprotein E-deficient mouse model of lipid metabolic disorder, the presence of NKG2D led to high production of inflammatory cytokines (such as IL-6, IL-12, and IFNγ) and the accumulation of NK cells, iNKT cells, and macrophages, resulting in a higher level of liver damage when compared to *Klrk1^−/−^* mice and to wild-type mice treated with NKG2D blocking antibodies (137). Vilarinho et al. developed a mouse model of acute hepatitis B generated through hepatic expression of small, middle, and large envelope proteins of hepatitis B (138). They demonstrated that the development of hepatitis resulted in an increase in mRNA and cell surface expression of RAE-1 on hepatocytes. Furthermore, hepatitis only occurred in the presence of functional B, T, and NKT cells and treatment with NKG2D neutralizing antibody dramatically reduced IFN-γ and IL-4 expression and liver damage. More recently, Huang et al. established a model of fulminant hepatitis induced by the double-stranded RNA mimic TLR3 agonist polyinosinic:polycytidylic acid in conjunction with the hepatotoxin d-galactosamine. In this model, mice treated with a plasmid coding for shRNA that inhibits the expression of all known mouse NKG2D ligands showed decreased liver damage and lower IFN-γ expression by NK cells, demonstrating that liver damage is reduced in the absence of ligands (139). In agreement with this, Chen and colleagues showed that RAE-1 and MULT1 expression on hepatocytes contribute to autoimmune liver injury similar to that observed in HBV-chronic infection (140). Using a model of Con A-induced hepatitis in the hepatitis B-Tg mouse, the authors showed an accumulation and activation of NK cells that could be prevented by blocking NKG2D (140).

The obvious commonality between these studies is the remarkably high level of expression of NKG2D ligands on hepatocytes, which suggests that the severity of hepatitis and consequent progression to HCC is dictated by the strength of the NKG2D response. A strong NKG2D-mediated immune response may be desirable to clear virally infected and transformed cells; however, it could be deleterious against uninfected hepatocytes causing more damage that sustain inflammation. Alternatively, downregulation of NKG2D and/or expression of NKG2D ligands with low binding affinity or at a low level may lead to a weak response unable to clear aberrant cells and consequently favors persistent infection associated with a smoldering inflammation that drives disease progression (92).

### Which Cell Types Are Involved in Tumor Rejection versus Promotion?

One key question relates to the cell type(s) involved in NKG2D-mediated promotion of tumor growth. The liver contains a large population of lymphocytes, including CD8^+^ T, CD4^+^ T, NK, and iNKT cells, all potentially activated *via* NKG2D in the DEN model. In accordance with previous work, we observed a remarkable enrichment in memory CD8^+^ T cells in the liver of wild type DEN-treated mice compared to age-matched non-treated control mice (136, 141). CD8^+^ T cells, of which at least a quarter expressed NKG2D, represent the main source of IFNγ (136). Evidence of NKG2D downregulation suggests that CD8^+^ T cells have been activated *via* NKG2D engagement in the TME and NTME. The idea that CD8^+^ T cells contribute to tumor growth in this model is counterintuitive because: (i) DEN-treated RAG-deficient mice develop liver tumor nodules earlier than WT mice, at 6 months of age, supporting the importance of adaptive immunity in preventing early tumor development (141) and (ii) high CD8^+^ T cell infiltration is a hallmark of so-called “hot” tumors, correlating with a greater antitumor response in various cancer types and with better responses to immunotherapy (142). Yet, in a large cohort of 302 HCC patients, the presence of CD8^+^ T cell alone did not correlate with overall survival or disease-free survival (143). In fact, a number of recent model studies point to a role for CD8^+^ T cells in accelerating liver damage and HCC. In a study of 63 HCV-infected HCC patients, Ramzan and colleagues reported that the number of tumor-infiltrating CD8^+^ T cells significantly correlated with higher tumor recurrence and decreased overall survival of cancer patients (144). In a model of hepatitis-induced HCC, Haybaeck and colleagues examined the role of lymphotoxin α and β and their receptors in tumor growth. Breeding a lymphotoxin-expressing transgenic mouse onto a RAG-deficient background reduced HCC formation, demonstrating a detrimental role of adaptive immunity in this model (145). Wolf et al. demonstrated the negative impact of CD8^+^ T cell infiltration and activation in the development of NASH-induced HCC. Using RAG-deficient, β2m-deficient mice and CD8^+^ T cell depletion in wild-type mice, they showed that the infiltration and activation of CD8^+^ T cells and NKT cells is directly linked to liver damage and subsequent HCC in this model (146). The critical role of lymphotoxin β and hepatic CD8^+^ T cell was further demonstrated by Endig et al. in a mouse model of chronic liver failure leading to HCC development (147).

The analogy between CD8^+^ T cell activation in HCC and in inflammatory disorders builds on increasing evidence for the presence of NKG2D ligands on healthy tissues (7, 80, 148) and their contribution to wound-associated inflammation (149) and autoimmune diseases (27, 127, 137, 150–152). The contribution of NKG2D in autoimmune attack has been shown against several tissues including the lung (153, 154), skin (149), pancreatic islets (150), brain (152), joints (27, 155), liver (137), and gut (127, 156–160).

With regards to the role of NKG2D in liver cancer, there is no direct evidence that NKG2D^+^ CD8^+^ T cells promote HCC, but robust data support a detrimental effect of NKG2D^+^ CD8^+^ T cells during viral hepatitis. In patients with chronic hepatitis B and C, intrahepatic CD8^+^ T cells display a significantly increased expression of NKG2D compared to healthy controls (112). In acute hepatitis A virus (AHA)-infected patients, virus-specific CD8^+^ T cells expressed significantly higher levels of NKG2D compared to healthy donors and MICA/B was overexpressed in infected liver tissues (161). This promoted innate-like CD8^+^ T cell cytotoxicity and consequently mediated host injury in AHA (161).

We noticed that a significant fraction of CD4^+^ T cells expressed NKG2D in DEN-induced HCC and that CD4^+^ T cells in both TME and NTME may be a significant source of IFNγ and to a lesser extent of IL-17 (unpublished). As demonstrated in multiple studies, CD4^+^ T cells could be a main contributor to the pro-inflammatory environment *via* IFNγ and IL-17 secretion, as seen in Crohn’s disease patients (151, 162) and other autoimmune conditions (27). Second, they could help recruit CTL to the liver. Using the RMA/S-Rae-1 transplantable tumor model, Westwood et al. showed that CD4^+^ T cells helped establish effective CD8^+^ T cell memory against re-challenge (163). Additionally, they could directly cause cytotoxicity against hepatocytes as previously shown in HCC (164) and against melanoma (165, 166). Finally, NKG2D^+^ CD4^+^ T cells could act as suppressive cells in NKG2D ligand-expressing late stage tumors *via* the release of soluble Fas ligand (167). In patients with chronic HBV infection, MICA/B was induced on a small fraction of intrahepatic activated CD4^+^ T cells, which correlated with an increased proportion of activated NK cells (115). It will be interesting to see whether this is the case in HCC patients. While we did not observe NKG2D ligand expression on any lymphocytes in the DEN model of HCC, we cannot rule out that this is occurring for a short period of time during tumor progression, despite it being no longer present at end point.

The role played by NKG2D on NK cells in the DEN-induced tumor model is unclear. NK cells represent a small fraction of lymphocytes residing in the liver of 15-month-old mice (less than 5%) and were not enriched in DEN-treated mice compared to untreated age-matched control mice, which is in line with the IL-1R8 dependent impairment of NK accumulation in this model (168). Nonetheless, the fraction of CD107a-positive NK cells capable of IFNγ-production in HCC tumors is neither lower nor higher than in young control untreated mice, suggesting that NK cells are functional to some extent and may contribute to inflammation by increasing pro-inflammatory cytokines (136, 168). NKT cells are a predominant cell type residing in healthy liver tissues in mice and known to act as effective antitumor effectors in HCC in mouse models (169). NKT cells are likely to play a central regulatory function in the DEN-induced tumor model, contributing to NK cell and CD8^+^ T cell recruitment and activation. NKT cells can also contribute to hepatitis and NKG2D-dependent liver damage in mouse models possibly *via* cytokine production and direct targeting of hepatocytes (138, 170).

### What Are the Mechanisms Involved?

The second key question relates to the function of NKG2D^+^ lymphocytes. In wild-type mice, does NKG2D expression sustain the inflammatory milieu that drives further liver damage? Or does chronic NKG2D engagement cause anergy in the lymphocytes upon which it is expressed, repressing their antitumor function?

#### Is There a Cell Subset With a Pro-Tumor Function?

A subset of TCRαβ^+^CD8^+^CD44^+^CD62^−^ T cells (T-pro) were shown to promote cutaneous carcinogenesis in the 7,12-dimethylbenz[a]anthracene (DMBA) and phorbol 12-myristate 13-acetate (PMA)-induced model of cutaneous carcinoma in a dose-dependent manner (171, 172). At high doses of DMBA in combination with PMA, the T-pro cells produced large amounts of IFNγ and TNFα and expressed elevated levels of NKG2D transcripts, but low amounts of perforin (172). In contrast, at low doses, the pro-inflammatory response was driven by a TH17-type response (173). In both cases, perforin was downregulated in the infiltrating CD8^+^ T cells, suggesting that this T cell subset with limited lytic activity promotes tumor growth *via* cytokine secretion. These findings are consistent with the proposed role of NKG2D in promoting inflammation-associated cancer *via* CD8^+^ T cells. Nonetheless, in the DEN-induced HCC model, CD8^+^ T cells produced IFNγ and TNF-α, but not IL-17; CD4^+^ T cells, however, were a potential source of IL-17 and could be acting as a subset with pro-tumor function *(unpublished)*.

#### Are NKG2D-Expressing Cells Activated or Desensitized *via* NKG2D Ligand Binding?

Sustained engagement of NKG2D by its ligands on tumor cells (174) or on myeloid cells can cause its downregulation and reduce NK cell responsiveness (76). Thompson et al. showed that RAE-1 expressed on lymph node endothelial cells and on tumor-associated endothelium can cause NKG2D internalization and desensitize NK cells. This led to higher tumor burden in models of subcutaneously transplanted tumors and in the transgenic adenocarcinoma of mouse prostate (TRAMP) model of prostate cancer (82). NK cells in NKG2D-deficient mice, due to the lack of NKG2D-mediated desensitization, appear more responsive as they are capable of a better tumor rejection compared to wild-type mice in the TRAMP model. This suggests that NK cell desensitization *via* NKG2D binding constitutes a mechanism of tolerance in wild-type mice (82). Whether RAE-1 is expressed on endothelial cells in the DEN-induced HCC model and whether RAE-1 on healthy hepatocytes can desensitize hepatic NK cells remains to be determined, but this is a plausible explanation for the maintenance of a tolerogenic liver NK cell phenotype at steady state. It is also an attractive explanation for the increase in tumor burden seen in DEN-treated wildtype compared to NKG2D-deficient mice (136). However, in the DEN model, NKG2D was expressed on all hepatic NK cells and its level of expression (based on fluorescence intensity of anti-NKG2D staining) was similar to levels detected in the naïve liver of control mice *(unpublished)*. In line with this, intrahepatic NK cells in HBV- and HCV-infected patients also maintain high levels of NKG2D expression during infection (113, 114). HCC patients showed high levels of cell surface NKG2D on liver-resident NK cells, although at a lower intensity on those infiltrating HCC compared to those residing in healthy tissue (175). Also, in the HBV model of acute hepatitis in transgenic mice developed by Vilarinho and colleagues, NKG2D was slightly downregulated on NKT cells found to be the main drivers of hepatitis, but not on NK cells (138). A possible explanation relates to the organization and location of NK cells in the diseased or tumor-bearing liver. In contrast with other CTL, there is no evidence to date that NK cells located in the liver sinusoids efficiently enter the space of Disse to make physical contact with hepatocytes (176, 177). If NK cells make contact with NKG2D ligand-expressing hepatocytes, it may not last long enough to induce NKG2D downregulation. Deguine et al. showed that NK cell interaction with transplanted RAE-1^+^ transfected EL4 tumor cells are more dynamic and transient that NKG2D^+^CD8^+^ T cells/tumor interactions (178). In support of this, we noticed that NK cells infiltrating DEN-induced HCC do not express PD-1 in contrast with CD8^+^ T cells, which may suggest a lack of chronic activation—assuming the mechanism of PD-1 expression is comparable in T and NK cells. Another possible explanation for the lack of NKG2D downregulation on NK cells and modest downregulation of NKG2D observed in CD8^+^ T cells in the DEN model relates to the presence of sMULT-1 in the serum of DEN-treated mice concomitant with high levels of MMP-9, MMP-14, and ADAM-10. As shown by Deng et al. (76), sMULT-1 could counteract RAE-1-mediated NKG2D downregulation in the DEN-induced HCC model by competitive binding to the receptor.

One limitation in our studies is that direct cytotoxicity of NK and CD8^+^ T cells against liver tumor cells could not be tested due to the difficulty of isolating viable hepatocytes for *in vitro* killing assay. Nonetheless, *ex vivo* analysis of CD107a expression by tumor-infiltrating CD8^+^ T cells showed that about a quarter of CD8^+^ T cells are surface CD107a^+^, which is higher than the frequency detected in non-treated liver controls, demonstrating that CD8^+^ T cells are degranulating in the tumor (136). Further supporting this, a larger fraction of CD8^+^ T cells produced IFN-γ in DEN-treated livers compared to young naïve mice (average 40 versus 20%) illustrating that these cells are responsive and that NKG2D downregulation in this model does not result in complete functional anergy (136). It is conceivable that NKG2D downregulation on CD8^+^ T cells is actually a sign of activation associated with endocytosis and signaling of the NKG2D/DAP10 complex (179). With regards to NK cells, the percentage and amount of IFN-γ^+^ NK cells in DEN-treated mice is similar to that observed in control naïve mice (136), suggesting that they are neither more active nor desensitized *via* NKG2D at this point of disease progression.

#### Does the Pro-Inflammatory Milieu Boost NKG2D-Mediated Responses?

In the DEN-induced HCC model, RAE-1 was not only expressed on transformed hepatocytes but also non-transformed hepatocytes in DEN-treated and age-match control mice compared to young controls, indicating that ligand expression increases with aging in the liver. This is probably due to the ongoing exposure to gut-derived microbial agents and/or to the occurrence of metabolic disorders consequent to weight gain over time.

The sustained expression of RAE-1 on tumors, i.e., lack of editing, and mild downregulation of NKG2D could be the key combination to sustaining a loop of liver damage as seen in autoimmune diseases. In support of this, one critical observation made in patients with various autoimmune diseases is the high level of NKG2D expression despite the presence of soluble ligands in the serum (27, 137, 156). In patients with rheumatoid arthritis, soluble MICA failed to downregulate NKG2D on CD4^+^ T cells and CD8^+^ T cells, possibly due to counteractions of IL-15 and TNF-α (27). The mild, rather than substantial, downregulation of NKG2D and lack of functional impairment of CD8^+^ T cells in the DEN-induced HCC model supports the idea that the inflammatory milieu in HCC, like in autoimmune diseases, thwart anergy. IL-15 present in the milieu is the best candidate to counteract NKG2D-mediated downregulation and NK cell and CTL desensitization as shown in autoimmune diseases and *in vitro* studies (58, 180, 181). Notably, under inflammatory conditions, the NKG2D-IL15 pathway leads to CTL infiltration and upregulation of NKG2D ligands associated with inflammatory myopathies (182). In rheumatoid arthritis, the substantial amount of soluble MIC released by synoviocytes failed to downregulate NKG2D on CD4^+^ T cells, possibly due to high levels of IL-15 and TNF-α (27). When exposed to IL-15 in culture, liver-resident NK cells isolated from HCC patients displayed increased NKG2D expression and functionality against cocultured targets (175).

In conclusion, we show that RAE1 and MULT1 are expressed in the DEN-induced HCC model, supporting the idea that NKG2D has been persistently engaged on CD8^+^ T cells leading to partial NKG2D downregulation from the cell surface. It remains to be determined whether NKG2D ligands expressed on hepatocytes or stromal hepatic cells contribute to maintaining a tolerogenic liver at steady state, an equilibrium likely lost in contexts of infection, sterile inflammation caused by obesity, or tumorigenesis.

### TME versus NTME: Does the Location of NKG2D-Expressing Cells Impact Their Function?

The ability for effector cells to migrate and reside in the TME is a key parameter in determining the positive or negative function of NKG2D expressing lymphocytes. CD8^+^ T cells have the ability to infiltrate the liver and establish contact with hepatocytes in healthy and infected tissues (176, 177) probing for cognate MHC/peptide on hepatocytes (183). In the DEN-induced HCC model, an enrichment of CD8^+^ T cells was observed in the NTME of wild-type mice compared to NKG2D-deficient mice, but not in the TME. This could be explained by a better recruitment and retention of CD8^+^ T cells in the NTME.

In support of this scenario, we found a greater amount of chemokines including CXCL9, CXCL10, CCL3, and CCL5 in the NTME of wildtype than NKG2D-deficient mice. These chemokines may be involved in CD8^+^ T cell chemotaxis to the DEN-treated liver and/or be produced by CD8^+^ T cells upon NKG2D-mediated activation in wild-type mice. Ligand expression may play a role in CD8^+^ T cell enrichment in DEN-treated liver. Indeed, transgenic RAE-1 expression on pancreatic islet cells was previously shown to favor the recruitment of CD8^+^ T cells *in vivo* during pancreatic inflammation where CCL5 was significantly elevated (184). Also, CD8^+^ T cells were activated and recruited in an NKG2D-dependent manner *in vitro* across a monolayer of ligand-expressing human intestinal endothelial cells (160). Furthermore, in the DEN model, we observed a significantly higher proportion of neutrophils in the NTME of wildtype compared to NKG2D-deficient mice supporting their role in NKG2D-mediated recruitment of CD8^+^ T cells to NTME (136). In a transgenic mouse model of HBV, neutrophils were critical for the recruitment of antigen non-specific T cells to inflamed liver (185). Neutrophils recruit CD8^+^ T cell due to their capacity to promote and amplify an initial inflammatory response by secreting chemokines, including CXCL9, CXCL10, CCL3, and CCL5 (186, 187). Overall, NKG2D contributes significantly to CD8^+^ T cell enrichment in the inflamed liver tissue adjacent to tumors, seemingly *via* increased chemotaxis of T effector memory cells (T_EM_) and persistence as tissue-resident T cells.

In addition to the above possibilities, it is conceivable that CD8^+^ T cells are found in higher amount in the NTME compared to the TME of wild type because they do not optimally infiltrate HCC tumors, regardless of NKG2D and NKG2D ligand expression. Due to inherent differences between the tumor tissue and the NTME, such as increased cell density and necrosis, it is possible that the ability of CD8^+^ T cells to traffic to or within the surrounding tissue is superior to their ability to infiltrate tumors. The vasculature of HCC is characterized by “disorganized, tortuous vessels” that decrease in number as histological grade progresses (188) and restrict T cell infiltration (189). Cytotoxic T cell density was shown to be markedly reduced in tumor-cell rich areas and most CTL were retained at the border of such regions by long-lasting contacts (190). Thus, NKG2D-mediated recruitment, effective in the NTME, may be inhibited by such contacts in the TME. We do not currently have further evidence to prove this hypothesis in the present model, protein expression of chemokines and chemokine receptors will have to be quantified and compared in wildtype and NKG2D-deficient mice.

#### Impact of NTME in HCC Progression

In HCC patients, the adjacent NTME has been described as the site of a pro-inflammatory immune response that enhances tumorigenesis (133). Hoshida and colleagues showed that pro-inflammatory gene expression (TNFα, IL-6, and nuclear factor-κB) in the NTME, rather than the tumor itself, strongly associated with poor prognosis and high recurrence rate in patients undergoing hepatic resection (133). Interestingly, a significant proportion of CD8^+^ T cells detected in the NTME of HCC patients are organized in microniches referred to as ectopic lymphoid-like structures (ELS). While the function of ELSs remains understudied, they are known to develop at sites of inflammation and signify good prognosis in certain malignancies. However, the formation of ELSs was shown to foster tumor growth in the DEN model of HCC (191). ELS development was abolished following T cell depletion and hepatocarcinogenesis was attenuated, demonstrating the importance of adaptive immune cells and the cytokine-rich microniche (191). ELS could have functional implications on the action of NKG2D in the DEN-induced HCC model, fostering NKG2D-mediated CD8^+^ T cell activation by ligand-expressing hepatocytes, which subsequently exacerbate proinflammatory responses in the NTME.

Hepatic stellate cells (HSC) are non-parenchymal cells that constitute 20–30% of the liver tissue and, along with Kupffer cells (KC), they contribute to liver pathogenesis due to their capacity to secrete various cytokines, recruit and activate lymphocytes including NKT and T cells. Interestingly, activated T cells can enhance HSC activation, resulting in IL-6, IL1-α, and TGFβ production (192), thus adding to the inflammatory milieu of cytokines promoting tumor growth.

#### Impact of TME in HCC Progression

**Figure 1:**
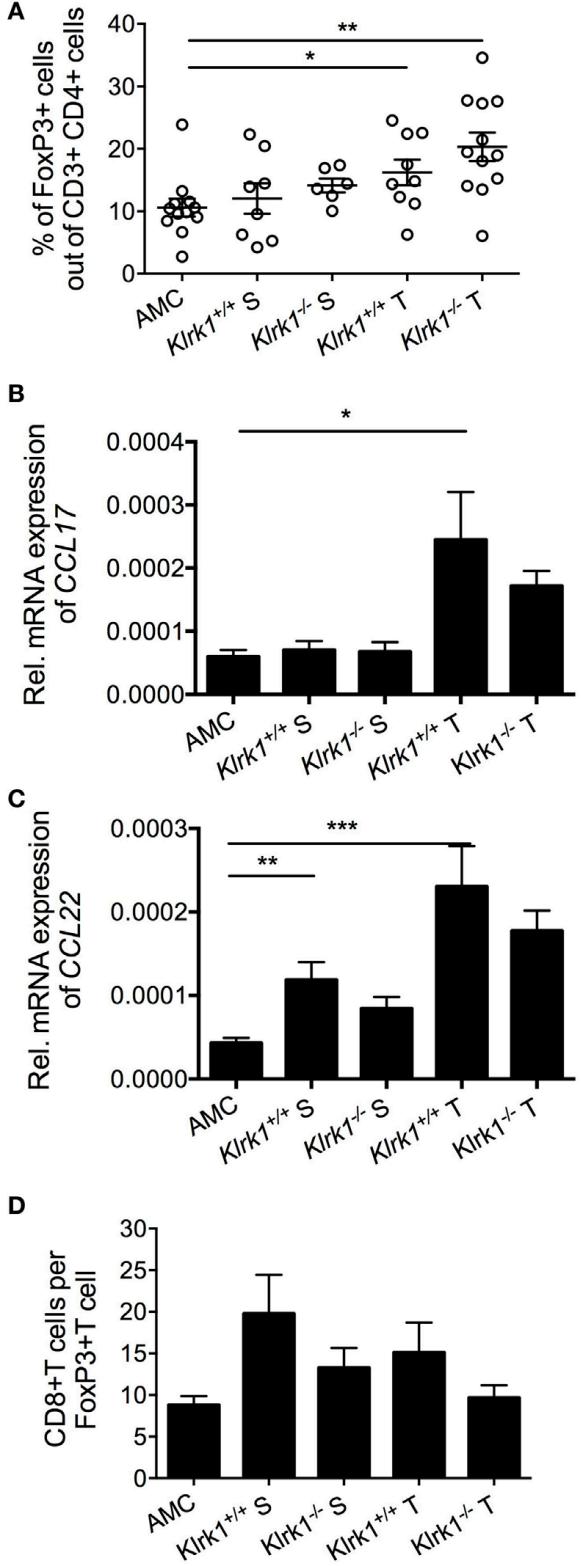
Accumulation of Foxp3^+^ CD4^+^ T cells in diethylnitrosamine (DEN)-treated liver. **(A)** Percentages of FoxP3^+^ T cells (gated on CD4^+^ cells) present within the tumor (T) and the surrounding tissue (S) in wildtype (*Klrk1^+/+^*) and NKG2D-deficient (*Klrk1^−/−^*) DEN-treated mice, and age-matched control mice (AMC). **(B,C)** Relative expression of **(B)** CCL17 and **(C)** CCL22 mRNA transcripts within tumors (T) and surrounding tissue (S) in wildtype and NKG2D-deficient DEN-treated mice (*n* ≥ 20) and age-matched control mice (AMC) (*n* ≥ 8). **(D)** Ratio of CD8^+^ T cells to FoxP3^+^ T cells in wildtype (*Klrk1^+/+^*) and NKG2D-deficient (*Klrk1^−/−^*) DEN-treated mice, and AMC. Graphs represent the mean ± SEM. Statistical analysis was performed by unpaired Student’s *t* test, where statistically significant differences between groups are denoted as: *p ≤ 0.05 and **p ≤ 0.01.

The TME is an immunosuppressive environment with the ability to impair antitumor responses. Transcriptional and epigenetic changes may underpin the generation of dysfunctional T cells in the TME. Single-cell RNA-seq revealed a distinct clonal population of exhausted CD8^+^ T cells that was elevated in late-stage HCC compared to early stage tumors associated with enrichment in regulatory T cells (Tregs) (193). Tregs were markedly increased in DEN-induced HCC compared to age-matched liver tissue (Figure 1A). This correlated with an increase in CCL17 and CCL22 transcripts (Figures 1B,C), two chemokines involved in Treg recruitment (194). In addition to the numerous mechanisms employed by Tregs to repress NK and CD8^+^ T cell antitumor functions, Tregs may play a role in the mild downregulation of NKG2D in CD8^+^ T cells through TGFβ production, as seen in patients with persistent HBV infection (92). It has been shown that adoptive transfer of Tregs into Rag1-deficient mice inhibited the NK cell antitumor function against RAE-1 expressing B16 cells, implicating an inhibitory role for Tregs in NKG2D-mediated surveillance (195) or NKG2D signaling (196). However, Tregs were remarkably less represented than CD8^+^ T cells in DEN-treated liver (Figure 1D), suggesting that Treg-mediated suppression of CD8^+^ T cell antitumor activity may not be effective in this model. Also, NKG2D downregulation potentially mediated by sustained binding to NKG2D ligands, soluble ligands, and/or TGF-β could be counteracted by cytokines such as TNF-α, IL-15, IL-2, and IL-18 present in the milieu (79, 197), which would explain the mild, rather than drastic, downregulation we observed.

In line with clinical studies showing significantly higher levels of PD-1^+^CD8^+^ T cells in diseased human liver compared to healthy liver tissue and peripheral blood (198), the majority of CD8^+^ T cells infiltrating DEN-induced tumors expressed PD-1. Interestingly, a majority of PD-1^+^CD8^+^ T cells were functionally active as determined by GzB expression and IFNγ production. It is conceivable that the fraction of PD-1^+^CD8^+^ T cells that do not express IFNγ are dysfunctional and have impaired antitumor activity. Encouraging results were obtained from a clinical trial (checkmate40) using nivolumab to inhibit PD-1 in the treatment of advanced HCC patients (199, 200). It is tempting to postulate that the unleashed antitumor response against tumor-associated antigens (TAA) in treated patients is enhanced through NKG2D costimulation of the TCR. There are indications that this may be the case, as the presence of human soluble NKG2D ligand has been shown to correlate with poor response to checkpoint blockade therapy (70), with Ab neutralization of NKG2D ligands showing the potential to enhance efficacy and reduce autoimmune side effects (201). Furthermore, NKG2D-dependent, TCR-independent stimulation may also contribute *via* the elimination of NKG2D ligand-expressing tumor variants that have lost TAA expression.

Myeloid cells present in the TME could also impede antitumor responses in the DEN model as seen in other models of cancer (83). Recent data obtained by Vyas et al. in ovarian cancer patients, a type of cancer with a pro-inflammatory signature similar to HCC, demonstrated that high levels of soluble MICA and ULBP2 were associated with poor prognosis. Interestingly, this did not correlate with NKG2D downregulation, but rather with reduced infiltration of effector memory CD8^+^ T cells and increase infiltration of pro-tumorigenic macrophages (74).

With regards to NK cells, there is some evidence, from preclinical and clinical studies, that the degranulation response of hepatic NK cells is impaired in the DEN model (168) HCC patients (175) and chronic HCV-infected patients (114) is reduced. NK cells from HCV infected patients degranulated less, but produced equivalent levels of IFNγ and TNFα, in anti-NKG2D mAb mediated redirected cytotoxicity experiments in the presence of IL2 and IL12 (114). This suggests that the cytotoxic capacity of intrahepatic NK cells in patients with chronic hepatitis C may be reduced. Additional evidence was obtained by incubating peripheral blood NK cells from healthy donors with an HCV-infected HCC cell line for 18 h, this reduced degranulation and IFNγ production against K562 cells (202).

### Are CD8^+^ T Cells Involved in Antigen-Specific or TCR-Independent Responses?

**Figure 2:**
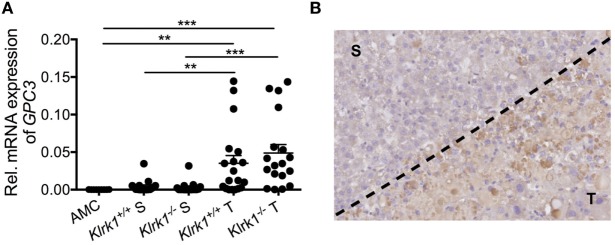
Tumor-specific expression of glypican3 in hepatocellular carcinoma. **(A)** Quantification of *Gpc3* mRNA transcripts within tumors (T) and surrounding liver tissue (S) in wildtype (*Klrk1^+/+^*) and NKG2D-deficient (*Klrk1^−/−^*) diethylnitrosamine (DEN)-treated mice (*n* ≥ 17) and AMC (*n* = 9). **(B)** Representative IHC staining of GPC3 on a DEN-treated liver where T represents the tumor area and S represents the surrounding tissue. Graph represents the mean ± SEM. Statistical analysis was performed by unpaired Student’s *t* test, where statistically significant differences between groups are denoted as: **p ≤ 0.01 and ***p ≤ 0.001.

In the DEN model, more than half of the memory CD8^+^ T cells infiltrating the tumors expressed PD-1, highlighting their prior encounter with antigens and chronicity of the TCR stimulation. This supports the idea that the majority of CTL in the TME are antigen-specific and may recognize a TAA still present on tumor cells. We detected a large amount of glypican 3 (GPC3)—one of the most frequently expressed known TAAs in human HCC (203)—suggesting that GPC3 could be responsible for CD8^+^ T cell infiltration and activation in the tumor bed (Figure 2) (136). Whether those cells are still functionally active in eliminating TAA^+^ tumors at the advanced stage of HCC in the DEN model (15-month end point) or whether they are ineffective against tumor variants that have lost TAA remains to be determined.

It is tempting to postulate the existence of a TCR-independent CTL response in the DEN-induced HCC model, especially in the NTME. NKG2D mainly acts as a co-stimulatory receptor on CD8+ T cells but can also act as a stimulatory receptor under certain circumstances (204–206). Markiewicz et al. showed that RAE1ε enhanced CTL IFN-γ secretion in response to IL-12 and IL-18 in the absence of antigenic stimulation (206). In human gut tissues, high expression of MICA can directly activate NKG2D in γδT cells and co-activate CD8^+^ T cells that constitute the intraepithelial lymphocytes (IEL). These effector cells were shown to target gut epithelial cells in a TCR-independent manner as well as *via* a gliadin antigen-dependent CD8^+^ T cell response (156). Such lytic activity has led to villous atrophy in celiac disease caused by IEL-mediated damage likely *via* NKG2D activation, in a TCR-independent manner (127). Other studies have shown a direct role for NKG2D in a TCR-independent activation of gut IEL (156) in the presence of high does IL-2 (204). In the human intestinal epithelium, the presence of IL-15 might promote NKG2D expression or lack of downregulation (180) and could also be favorable to NKG2D signaling independently of the TCR (127). The established link between NKG2D and IL-15R signaling (207) led us to hypothesize that NKG2D-mediated activation of CTL in contact with NKG2D ligand-expressing normal hepatocytes, in the presence of IL-15, could lead to a self-damaging response in a non-cognate fashion. TCR-independent, NKG2D-dependent activation of memory CD8^+^ T cells was recently demonstrated in hepatitis A virus-infected (HAV) patients where liver injury associates with high production of IL-15 and elevated expression of NKG2D ligands by infected hepatocytes (161). The severity of liver injury in these patients correlated with the activation of HAV-unrelated virus-specific CD8^+^ T cells (specific for influenza A, Epstein–Barr, and cytomegalovirus) and the innate-like cytolytic activity of CD8^+^ T cells, but not the activation of HAV-specific T cells (161). It was also shown that coculture of peripheral CD4^+^NKG2D^+^T cells from metastatic melanoma patients with sMICA + IL-15 induced IFNγ secretion in a TCR-independent manner (49).

The importance of antigenic recognition in CTL migration and location in the TME has been highlighted in tumor transplant models (208) showing that CTL were able to infiltrate tumors in depth only if tumors displayed cognate antigens; otherwise, they remain in peripheral regions (209). We, therefore, propose that when there is a significant influx of tumor-specific CTL into an inflamed liver, only tumor-antigen-specific T cells infiltrate the tumor, possibly becoming anergized through persistent TCR engagement.

### Adverse Effects of NKG2D in HCC: Working Model

**Figure 3:**
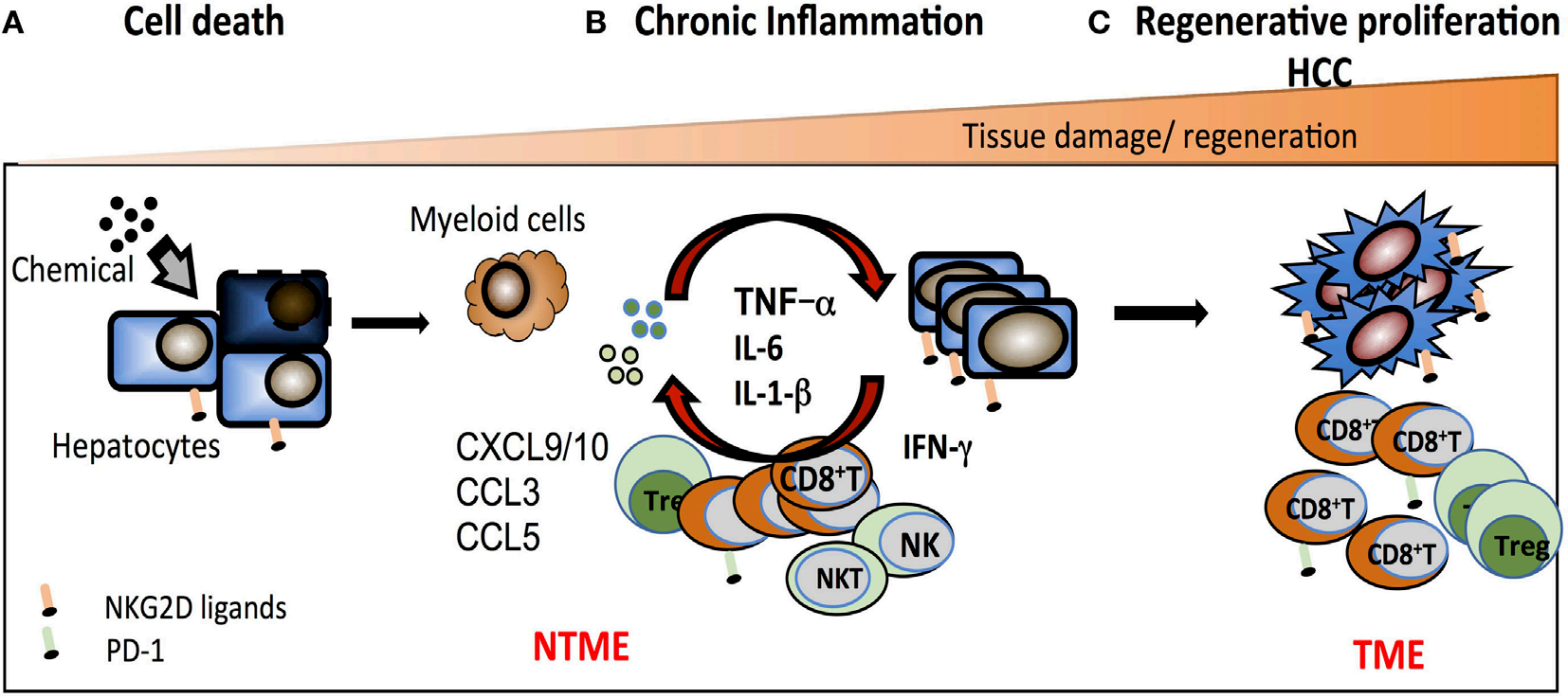
Working model of the pro-tumorigenic effect of NKG2D in hepatocellular carcinoma. **(A)** Chemical and/or viral injury causes cell stress that results in DNA damage, increased expression of NKG2D ligands, and hepatocyte cell death. NKG2D^+^-resident effector cells [natural killer (NK) cell, NKT cell, CD8^+^ T cells] participate in the elimination of stressed hepatocytes and contribute to shaping an innate inflammatory environment that further drives the recruitment and activation of antigen-specific CD8^+^ T cells. **(B)** In response to a continuous exposure to NKG2D ligands in the non-tumor microenvironment (NTME), NKG2D^+^ effector cells exacerbate the local inflammation *via* direct secretion of inflammatory components (such as TNFα, IFNγ, and MIP1-α) and chemoattractants leading to an enrichment in CD8^+^ T cells. This indirectly favors the recruitment of myeloid cells that further the inflammatory milieu with pro-inflammatory cytokines such as IL-6, TNFα, and IL-1α. **(C)** In this context, cycles of persistent tissue injury characterized by the death of hepatocytes consequently drives the process of tissue regeneration, which encompass the proliferation of mutated hepatocytes or epithelial cells and ultimately promotes tumor growth. In the TME, the majority of CD8^+^ T cells expresses PD-1 and may become partially impaired in their antitumor function due to antigen-specific chronic stimulation.

We postulate that early stage precancerous liver lesions are rejected *via* activation of NK and NKT cells through NKG2D engaging NKG2D-ligand expressing neoplastic hepatocytes. Over time, sustained tissue damage initiated by the mutagen creates an environment enriched in myeloid cells and chemokines such as CXCL9, CXCL10, CCL3, CCL5, and Mip1-a, which drive the recruitment of memory CD8^+^ T cells and inflammatory macrophages. Elevated perforin and granzyme B coupled with metalloproteinase activity amplifies the immune response and promotes liver injury. MP can also contribute to the shedding of ligands such as sMULT1, detected in HCC-bearing mice, that can counteract RAE-1-mediated desensitization. Continuous exposure of T cells to NKG2D ligands feeds the pro-inflammatory milieu *via* TNFα and IFNγ production in a TCR-independent, NKG2D-dependent fashion. Persistent hepatic injury, hepatocyte death, and regeneration of mutated hepatocytes ultimately results in tumor growth. This NKG2D-mediated inflammatory response is likely beneficial in rejecting early neoplastic tumors, yet, over time, feeds the loop of tissue injury-repair-proliferation that are the hallmark of HCC. In addition, the build-up of an immunosuppressive milieu within the TME and high levels of PD-1/PD-L1 expression is likely to impair tumor clearance by NK cells, NKT cells, and TAA-specific CTL (Figure 3).

### Tumor-Promoting Effect of NKG2D in Other Cancer Models

Previous studies have described how other immune components can act as antitumor versus pro-tumor effectors in the same model. For example, MyD88 was shown to promote tumor growth in the MCA-induced fibrosarcoma model, while simultaneously mediating a protective host response *via* TNFα and IFN-α/β signaling (210). IL-1α and IL-1β showed opposite functions in the development of fibrosarcomas induced by MCA. IL-1β acted as driver of oncogenic inflammation, IL-1β-deficient mice developed less tumors due to reduced pro-tumorigenic inflammation compared to wild-type mice. In contrast, IL-1α acted as a key mediator of immune surveillance; tumor cell lines derived from IL-1α-deficient mice showed reduced invasiveness when transplanted in wild-type hosts, demonstrating a lack of tumor editing in its absence. Loss of tumor editing in IL-1α-deficient mice was attributed to impaired NK cell maturation and a suboptimal killing capacity of effector immune cells resulting in reduced tumor editing (211). Also, αβ T cells have been shown to contribute to skin papilloma formation, reinforcing the idea that adaptive immunity plays a dual role in models of chemically induced inflammation and tumorogenesis (171, 212).

In a BALB/c mouse model of chronic pulmonary inflammation that progresses to lung adenocarcinoma due to the concomitant lack of IFN-γ and the β-common cytokines GM-CSF and IL-3, Dougan and colleagues showed that IL-6 is a key driver of oncogenic inflammation (213). These mice are also highly susceptible to tumor growth due to the lack of IFN-γ-driven tumor editing as demonstrated by their clearance upon transplant into immune-competent hosts. This model demonstrates the importance of IFNγ-driven tumor editing even in inflammation-driven cancer (213).

With regards to the NKG2D axis, tumor progression rather than rejection consequent to NKG2D ligands expression has been observed in other models of cancer. Our group has reached similar conclusions in a mouse model of intestinal polyposis and colon carcinoma. In this model, RAE-1 is expressed on the gut epithelium and associates with a significantly reduced survival of wildtype compared to NKG2D-deficient littermates *(unpublished data)*. In the TRAMP model, some TRAMP mice developed large, rapidly growing aggressive tumors (type I) that were rejected *via* NKG2D (35). However, some TRAMP mice developed a second type of tumor (type II) that progressed much slower, expressed high amounts of cell surface NKG2D ligands but were not rejected by NKG2D. Type II tumors actually grew faster in the presence of NKG2D, though the trend was not statistically significant. Although the TRAMP tumor milieu has not been extensively studied, an obvious difference between these tumors is that type II tumors displayed a high immune infiltrate that was almost non-existent in type I tumors *(unpublished)*. The presence of soluble NKG2D ligands was not tested, hence their expression cannot be ruled out and may differentially impact the control of type I versus type II tumors (36). Another model worth discussing is the MCA-induced fibrosarcoma where MyD88-dependent inflammation was shown to promote tumorigenesis (210). We observed that slow-growing tumors induced by a low dose of MCA (5 µg) expressed high levels of cell surface NKG2D ligand and showed a trend to develop earlier in NKG2D-sufficient than NKG2D-deficient mice (35). This was not the case in fibrosarcoma induced with higher dose of MCA (25 µg), which progressed significantly faster regardless of NKG2D (35). The high level of ligand expression and lack of editing in both TRAMP type II tumors and fibrosarcoma supports the idea of a tumor-promoting effect through NKG2D activation. Further studies to characterize the TME and NTME and eventual release of soluble ligands would be valuable. Collectively, these studies indicate that the extrinsic pro-tumor effect associated with NKG2D–NKG2D ligand signaling is not restricted to HCC and could broadly apply to cancer driven by chronic inflammation. Dissecting the function of NKG2D on CD8^+^ T versus NK versus NKT cells in this model will be the key to fully understand the function of NKG2D in inflammatory cancer. Experimental approaches that involve antibody depletion and/or adoptive transfer of specific cell types will be critical to this end in addition to the more definitive approaches of using conditionally genetically targeted mice.

## Concluding Remarks

**Figure 4:**
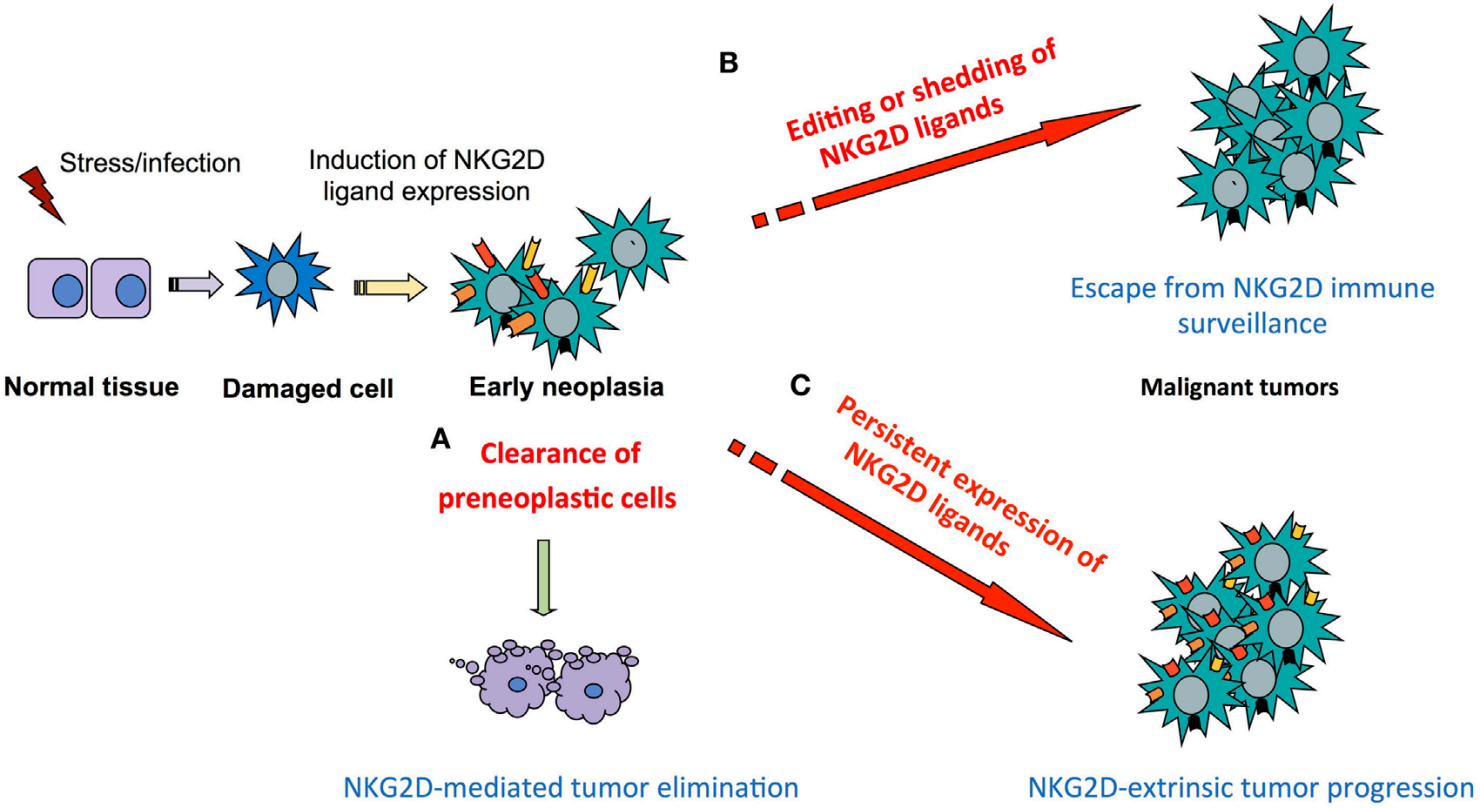
Principles of NKG2D dual function in tumor immunity. Induction of NKG2D ligand on stressed or damaged tissues initiates a local immune response *via* NKG2D-expressing cells that progresses according to four non-exclusive scenarios. **(A)** Clearance of neoplastic cells preventing or delaying tumor formation as seen in mouse models of Eμ myc driven B-cell lymphoma and leukemia and aggressive prostate tumors in the transgenic adenocarcinoma of mouse prostate (TRAMP) mouse model. **(B)** Failure to eliminate tumors due to NKG2D ligand editing as seen in a fraction of aggressive prostate tumors in the TRAMP mouse model or to ligand shedding and subsequent NKG2D downregulation and effector desensitization, as described in advanced human cancer with poor prognosis. **(C)** Persistent expression of NKG2D ligands at high levels exacerbates the pro-tumorigenic milieu *via* NKG2D-mediated immune responses in models of inflammation-driven cancer such as hepatocellular carcinoma and fibrosarcoma models.

In line with clinical studies that correlate high levels of NKG2D ligands with poor prognosis (43, 53–55), we observed in some mouse models that tumors were not efficiently rejected and even progressed in NKG2D-sufficient mice, despite high levels of surface NKG2D ligands expression on the tumors (35, 136, 214). Our studies of the DEN-induced model of HCC demonstrate a paradoxical role of the NKG2D axis in promoting tumor growth. NKG2D-mediated antitumor activities may exist in this model and we argue that antitumor and pro-tumor effects *via* NKG2D may occur in a time- and context-dependent manner (Figure 4). We propose that during HCC progression in DEN-treated mice, NK cells and NKT cells, *via* NKG2D, are likely to act as the main effectors against early neoplasia, although falling short of complete tumor elimination. Subsequently, CD8^+^ T cells may represent the main drivers of the pro-tumor effect, as seen in chronic inflammatory diseases and recently in HCC promoting inflammation in the NTME in an NKG2D-dependent, TCR-independent manner. Concomitantly, persistent expression of PD-1 on memory CD8^+^ T cells in the TME, along with other immunosuppressive components and tumor-intrinsic features, could reduce and/or prevent antigen-specific antitumor activity of CD8^+^ T cell.

There is no doubt about the potential of NKG2D/NKG2D ligands as targets in immunotherapeutic strategies. To deliver an effective and tailored NKG2D-based therapy, several questions remain to be addressed regarding the influence of the TME and the impact of radiotherapy and chemotherapeutic agents on NKG2D ligand expression—as these could potentially impair or eventually worsen the treatment. The kinase inhibitors Sorafenib and recently approved Regorafenib, used as second line treatment against advanced HCC, offer a limited survival benefit to advanced HCC patients. With the recent FDA approval of the anti-PD-1 mAb nivolumab for treatment of HCC, one might hope to specifically target tumor-infiltrating CD8^+^ T cells and potentiate tumor antigen-specific response in the TME, including NKG2D-mediated surveillance, without enhancing the detrimental pro-inflammatory effect of NKG2D in the adjacent healthy tissue.

The finding that cancer may progress as an adverse effect of high expression of NKG2D ligands and NKG2D activation represents a challenge to the design of NKG2D-based immunotherapies due to the potential toxicity against healthy tissues expressing NKG2D ligands (215, 216). Approaches aiming at enhancing NKG2D ligands expression could contribute to the deleterious cycles of tissue damage and repair known to favor tumor growth over rejection in inflammation-driven cancer. Several clinical trials testing NKG2D-based chimeric antigen receptor (CAR) have been initiated; a first report of objective response to NKG2D-CAR (CYAD-01) was recently described in a relapsed/refractory acute myeloid leukemia patient (217). It will be interesting to see what type of solid tumors may benefit from this approach since it has the capacity to also target NKG2D ligand^+^ immunosuppressive cells present in the TME. Advanced HCC and other types of cancer associated with chronic inflammation may require NKG2D-based approaches that target tumor cells with minimal damage to the NTME. Alternatively, blocking NKG2D as a means to reduce hepatic chronic inflammation is an approach worth considering during hepatitis and is currently being tested in Crohn’s disease (159).

## Materials and Methods

### Mice

*Klrk1^−/−^* mice on a C57BL/6J background were bred and maintained at the Imperial College London’s animal facility, in a specific pathogen-free environment. The health status was regularly monitored throughout the study.

### HCC Induction

Cohorts of male age-matched wildtype (*Klrk1^+/+^*) and NKG2D-deficient (*Klrk1^−/−^*) mice received a single intraperitoneal injection of DEN (Sigma) (25 mg/kg body weight) or PBS at 14–21 days of age to induce HCC. Mice were euthanized at 15 months of age. Mice that became terminally ill prior to the endpoint were humanely sacrificed.

### Histology and Immunohistochemistry

Liver tissues were fixed in 10% neutral buffered formalin, paraffin embedded, and cut into 4 µm sections. Glypican-3 (GPC3) expression was analyzed using a rabbit polyclonal antiserum raised against GPC3 (ab66596) (Abcam, Cambridge, UK). Slide images were captured using a Nanozoomer slide scanner (Hamamatsu) and analyzed using Image J software.

### Tissue Dissociation and Flow Cytometry

Tumor and surrounding regions were delineated macroscopically. Tissues were dissociated through 100 µm cell strainers in PBS with 3% bovine serum albumin (BSA). Hepatocytes were removed by centrifugation on a 35% Percoll gradient at 700 × *g* at 21°C for 12 min. Leukocytes present in the pellet were resuspended in red blood cell lysis buffer (0.15 M NH_4_Cl, 0.1 mM KHCO_3_, 0.1 mM Na_2_-EDTA in water; pH 7.2) for 1 min, washed, and then resuspended in PBS with 3% BSA. Cell suspensions were incubated with anti-mouse CD16/CD32 (Becton Dickinson, BD, USA) to block Fc receptors and Fixable Viability Dye eFluor 506 (eBioscience, San Diego, CA, USA). Cells were then stained with a cocktail of directly conjugated mAbs [CD3 (17A2) BD, CD4 (RM4-5) eBioscience, CD8 (53-6.7) BioLegend, CD45 (30-F11) BioLegend and Foxp3 (NRRF-30) eBioscience] for 30 min at 4°C. Intracellular staining was performed with a transcription factor staining buffer set from (eBioscience). The relevant fluorescence-minus-one labeling conditions including the appropriate isotype-matched mAb were used as controls. All samples were acquired on an LSR Fortessa flow cytometer (BD) and analyzed with FlowJo version 9.3.1 or above (Tree Star, Ashland, OR, USA).

### RNA Isolation and Quantitative RT-PCR

Tumor and surrounding liver tissue were collected in RNA-later (Sigma) and stored at −80°C as per the manufacturer’s instructions. RNA was extracted using Qiagen’s RNeasy kit (Hilden, Germany) and reverse transcribed into cDNA with High capacity cDNA RT kit (Life Technologies). For some genes, an amplification step was performed using the TaqMan PreAmp Master Mix Kit (Applied Biosystems). Quantitative real-time PCR was carried out using the TaqMan system (Applied Biosystems), all values were normalized to GAPDH expression.

### Statistical Analysis

Statistical analyses were performed using GraphPad Prism software version 5.03 (GraphPad Software Inc.). Two-tailed unpaired Student’s *t*-test was performed with Welch’s correction when appropriate. Differences at *p* ≤ 0.05 were considered significant.

## Ethics Statement

All animal work was carried out in compliance with the British Home Office Animals Scientific Procedures Act 1986 (Project license number 70/7129).

## Author Contributions

JG and SS performed the experiments and analyzed the data. SS and AF wrote the manuscript. NG designed and supervised the studies, analyzed the data, and wrote the manuscript.

## Conflict of Interest Statement

NG receives funds from AstraZeneca. All other authors declare that the research was conducted in the absence of any commercial or financial relationships that could be construed as a potential conflict of interest.

## Abbreviations

HCC, hepatocellular carcinoma; MICA/B, major histocompatibility class I-related chain; RAE-1, retinoic acid early inducible gene-1; MULT1, murine UL16-binding protein-like transcript 1; MMP, matrix metalloproteinase; TRAMP, transgenic adenocarcinoma of mouse prostate; TME, tumor microenvironment; ULBP, UL16-binding protein; DEN, diethylnitrosamine; TIL, tumor-infiltrating lymphocyte; TRAIL, TNF-related apoptosis-inducing ligand; MCA, methylcholanthrene.
